# Transgenic *Schizochytrium* as a Promising Oral Vaccine Carrier: Potential Application in the Aquaculture Industry

**DOI:** 10.3390/md22120555

**Published:** 2024-12-12

**Authors:** Ke Ma, Lei Deng, Yuanjie Wu, Yuan Gao, Jianhua Fan, Haizhen Wu

**Affiliations:** 1 State Key Laboratory of Bioreactor Engineering, East China University of Science and Technology, Shanghai 200237, China; 2 Department of Applied Biology, East China University of Science and Technology, Shanghai 200237, China; 3 Biopharmaceuticals R&D Department, Ningbo Sansheng Biological Technology Co., Ltd., Ningbo 315000, China

**Keywords:** *Schizochytrium limacinum*, fructose-1,6-bisphosphate aldolase, *Edwardsiella tarda*, oral vaccine, aquaculture, functional feed, immunoprotection

## Abstract

*Schizochytrium limacinum* SR21, a kind of eukaryotic heterotrophic organism rich in unsaturated fatty acids, is an emerging microbial alternative to fish oil. The dietary inclusion of 15% SR21 was optimal for the growth performance of zebrafish. Previous studies demonstrated that fructose-1,6-bisphosphate aldolase (FBA) of *Edwardsiella tarda* is a valuable broad-spectrum antigen against various pathogens in aquaculture (e.g., *Aeromonas hydrophila*, *Vibro anguillarum*, *Vibro harveyi*, *Vibro alginolyticus*). We pioneered the development of stable *S. limacinum* SR21 transformants expressing the antigen protein FBA, exploring their potential as a novel oral vaccine for the aquaculture industry. The model animal zebrafish (*Danio rerio*) and ornamental fish koi carp (*Cyprinus carpio* koi) were harnessed to assess the immunoprotective effect, respectively. According to the quantitative expression analysis, zebrafish fed with recombinant *Schizochytrium* expressing FBA exhibited specific immune responses in the intestine. The expression levels of *MHC-I* and *MHC-II*, involved in cell-mediated adaptive immune responses, were significantly upregulated on the 14th and 28th days post-immunization. Additionally, the expression of highly specialized antibody genes *IgZ1* and *IgZ2* in mucosal immunity were significantly triggered on the 14th day post-immunization. Feeding koi carp with recombinant *S. limacinum* SR21-FBA increased the production of myeloperoxidase and FBA-specific antibodies in the sera. Furthermore, the sera of koi fed with recombinant *S. limacinum* SR21-FBA exhibited significant bactericidal activities against pathogen *E. tarda*. Thus, *S. limacinum* SR21 is a natural and highly promising oral vaccine carrier that not only provides essential nutrients as a functional feed ingredient but also offers specific immune protection to aquatic animals. This dual application is vital for promoting the sustainable development of the aquaculture industry.

## 1. Introduction

At present, the aquaculture industry relies heavily on fish oil derived from caught fish as the primary source of unsaturated fatty acids in fish feed. This reliance undoubtedly limits sustainable development. In recent years, with the increasing demand for sustainable alternatives, lipid-rich microorganisms have been gradually developed as potential alternatives to fish oil [1]. Among these microorganisms, *Schizochytrium* sp. stands out as a typical representative of industrialized fermentation for bio-oil production due to its light-independent growth and easily scalable fermentation process [2,3,4].

*Schizochytrium* sp. is a unicellular, oleaginous microbe that originates from marine environments. It was once thought to be a heterotrophic marine eukaryotic microalga [5]. This marine organism is rich in bioactive compounds, particularly docosahexaenoic acid (DHA), eicosapentaenoic acid (EPA), squalene, and carotenoids, which are valuable in the fields of nutritional foods, bioenergy, and aquaculture feed [6]. As is well known, omega-3 polyunsaturated fatty acids (PUFAs) are important for the health of aquatic animals [7]. Dietary inclusion of this microorganism not only contributes to weight gain and feed conversion rates but also supports the early-development of aquatic animals, enhancing their immune capabilities. Moreover, *Schizochytrium* sp. effectively ameliorates the lipid metabolism and improves the nutritional composition in aquatic animals, promoting their health status [8,9]. In our previous research, the dietary inclusion of *Schizochytrium* sp. in the feed significantly improved the growth performance of zebrafish, as well as their immune status and gut microbiota [10]. These findings underscore the potential and value of *Schizochytrium* sp. as an excellent feed additive, holding substantial implications for the advancement of the aquaculture industry.

Currently, the genetic modification of *Schizochytrium* sp. mainly focuses on enhancing the metabolic pathways of fatty acids to promote the accumulation of bioactive lipids [3,4,6]. However, there has been no quantitative analysis of the relevant enzyme proteins. Despite some progress in decoding the genome of *Thraustochytrium* spp., the application of genetic engineering in *Schizochytrium* sp. is hindered by the lack of standardized genetic manipulation techniques and novel genetic tools. This limits the application of this microorganism for recombinant protein expression [11]. Ramos-Vega et al. have reviewed the potential of *Schizochytrium* sp. in vaccinology and placed it in perspective [12]. Regarding the development of oral vaccine using *Schizochytrium* sp. ATCC20888 as a carrier, there have been some initial successes, particularly in mouse models where the expected immune responses have been triggered [13]. However, the successful development of oral vaccines for aquatic animals using *Schizochytrium* sp. has not yet been reported.

Fructose-1,6-bisphosphate aldolase (FBA) is an enzyme widely present in various organisms and primarily involved in the glycolytic pathway. Notably, this critical housekeeping enzyme can be secreted into the extracellular environment. In some pathogens, extracellular or surface-associated aldolases exhibit multiple ‘moonlighting’ functions, such as adherence, plasminogen binding, invasion, colonization, and immune evasion to host cells, which enhance the virulence and survival capabilities of the pathogens [14,15,16,17,18]. As a common model organism, zebrafish are usually used in vaccine efficacy evaluations [19,20]. In our previous research, we utilized a reverse vaccinology approach to identify potential vaccine targets for the pathogen *E. tarda*, which is prevalent in aquaculture. Several enzymes related to the glycolytic pathway were found to be secreted extracellularly. Animal experiments confirmed that these purified enzymes could provide protection against pathogens in fish following injection immunization [21]. Among them, FBA, as a highly conserved representative across various aquaculture pathogens, has emerged as a potential and valuable candidate antigen target for a broad spectrum of pathogens [22].

It is worth mentioning that some probiotics, such as *Lactococcus* and *Lactobacillus* and *Bacillus subtilis*, have been used as oral vaccine vehicles to present antigens [23]. In this research, we first successfully utilized the edible and nutritional microorganism *S. limacinum* SR21 as a vaccine vector, developing a novel recombinant oral vaccine for the aquaculture industry. To improve the utilization efficiency of *S. limacinum* SR21 in feed, we evaluated the growth performance and resistance to the pathogen in zebrafish with different concentrations of SR21 powder. We then constructed a recombinant *S. limacinum* SR21 that expresses the antigen protein FBA from the pathogen *E. tarda* EIB202. To evaluate the efficacy of oral immunization with the recombinant *S. limacinum* SR21, zebrafish and koi carp were employed as experimental animals for a series of immunization assessments. We assessed the expression of immune-related genes and protection against the pathogen in zebrafish. Additionally, we determined the humoral immune responses in sera of koi carp.

## 2. Results

### 2.1. Determination of the Optimized Proportion of Schizochytrium in Feed

To improve the utilization efficiency of *S. limacinum* SR21 in feed, the optimal proportion of SR21 was determined. The initial weight, final weight, and survival rate of zebrafish in each group during the 4-week feeding experiment are shown in Table 1. The final weight of the fish in the 15% SR21 group was the highest among all groups. There were no deaths among the fish fed with different concentrations of SR21 and the fish exhibited high feeding enthusiasm.

**Figure 1:**
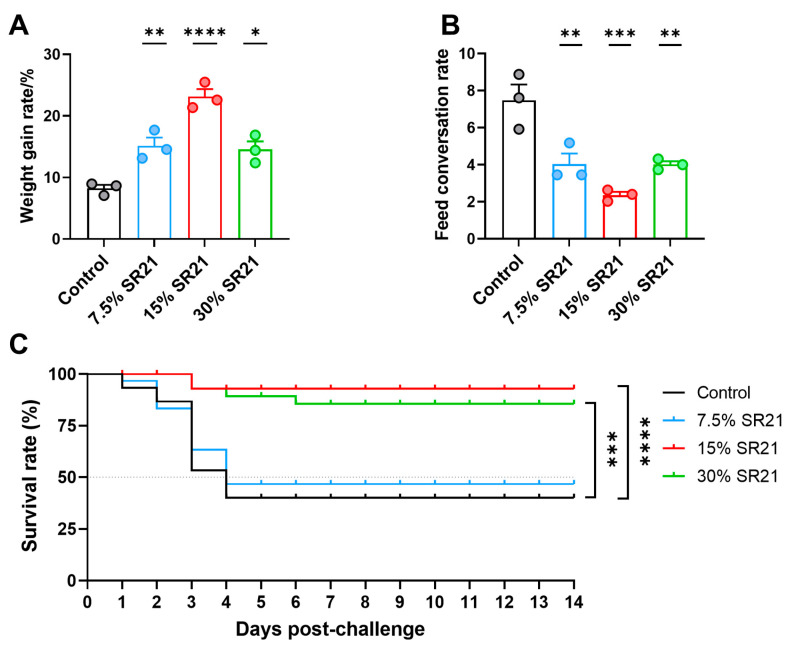
The growth performance parameters and resistance to pathogens of zebrafish fed with a gradient of *S. limacinum* SR21. Weight gain rate (**A**) (*n* = 3 pools, 10 fish per pool) and feed conversation rate (**B**) (*n* = 3 pools, 10 fish per pool); data are presented as the means ± SD. * *p* < 0.05, ** *p* < 0.01, *** *p* < 0.001, **** *p* < 0.0001. Survival curve (**C**) of zebrafish after immersion challenge with *V. anguillarum* MVM425 (3 × 10^6^ CFU/mL (CFU, colony-forming units)) (*n* = 30 per group); statistical differences were evaluated by log-rank (Mantel–Cox) test, *** *p* < 0.001, **** *p* < 0.0001.

The growth performance of zebrafish was evaluated after feeding them a gradient of *Schizochytrium*. Figure 1 shows the weight gain rate (WGR, Figure 1A) and feed conversation rate (FCR, Figure 1B) in each treatment group. Compared with the control group, WGR in all SR21 groups was significantly increased (7.5%, *p* < 0.01; 15%, *p* < 0.0, 001; 30%, *p* < 0.05). The WGR was highest in the 15% SR21 group among the four groups. In contrast, the FCR in all SR21 groups was significantly lower than in the control group (7.5%, *p* < 0.01; 15%, *p* < 0.001; 30%, *p* < 0.01), with the lowest FCR observed in the 15% SR21 group.

With respect to the protection against *V. anguillarum* MVM425 challenge, all SR21 groups exhibited varying degrees of protective effects (Figure 1C). Compared with the control group, the final survival rate was significantly higher in the 15% SR21 (*p* < 0.0001) and 30% SR21 (*p* < 0.001) groups. Typical clinical symptoms were apparent in dying fish, including a blackened body, erratic swimming, petechial hemorrhage, and lingering near the water’s surface. Notably, these clinical symptoms were delayed in the 15% SR21 and 30% SR21 groups.

### 2.2. Construction of Recombinant Schizochytrium Strains Expressing Antigen Protein FBA

**Figure 2:**
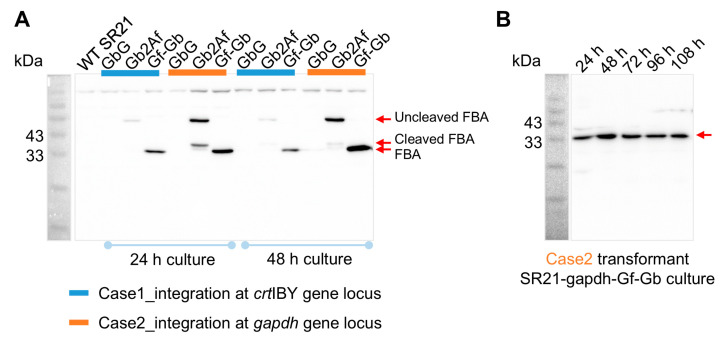
Western blot analysis of FBA expression. Expression of FBA in different *Schizochytrium* strains (**A**) and the SR21-gapdh-Gf-Gb strain at various cultivation time points (**B**). GbG, control check strain without antigen gene *fba*. G2Af, a GAPDH promoter, drives the co-expression of the marker gene *Ble* and the antigen gene *fba* (linked by a 2A sequence). Gf-Gb, a GAPDH promoter, solely drives the expression of the antigen gene *fba*.

We fused the antigen gene *fba* with a resistance gene through a 2A linking peptide element, thereby achieving the co-expression of both (Gb2Af transformants). Additionally, we constructed a separate expression cassette that exclusively contained the antigen gene *fba* (Gf-Gb transformants) (refer to Supplementary Data Figure S1). Through these methods, we aimed to screen the transformants that efficiently express the antigen protein. After selection, we obtained two types of recombinant SR21 strains with distinct integration sites: the antigen gene expression cassette integrated at the *crtIBY* and *gapdh* gene loci of the SR21 genome, respectively. The transformants with the cassette integrated at the *gapdh* locus exhibited a significantly higher expression level of the FBA antigen protein compared to those at the other integration site, as shown by Western blot analysis (Figure 2A). Fortunately, the transformant SR21-gapdh-Gf-Gb, which contains the sole *fba* expression cassette, exhibited the highest level of antigen protein expression. Moreover, we noted that the 2A linking peptide did not achieve complete self-cleavage, which might have implications for the expression and functionality of the antigen protein. Additionally, we conducted stability tests on the transformant SR21-gapdh-Gf-Gb and the results indicated that the antigen protein expression level remained relatively stable over extended culture periods (Figure 2B).

### 2.3. Oral Administration with Edible Schizochytrium-Based Vaccine in Zebrafish

**Figure 3:**
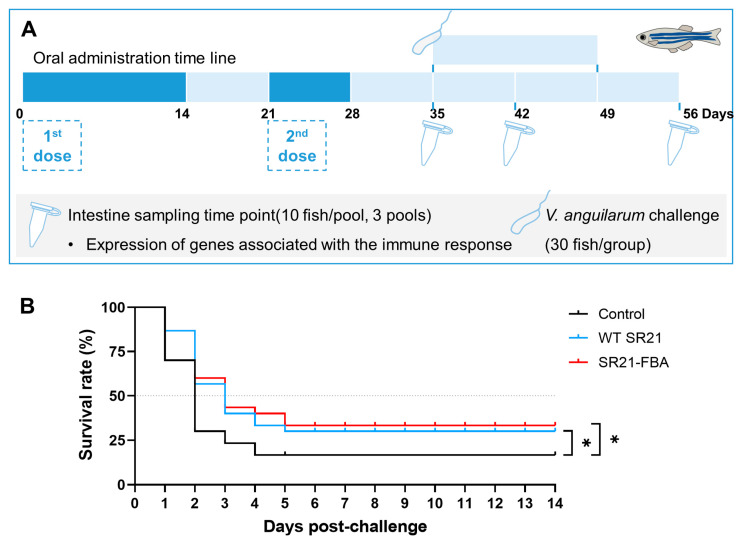
Two-dose immunization experiment in zebrafish. Zebrafish experimental scheme diagram (**A**) and survival curves of zebrafish challenged with *V. anguillarum* MVM425 (**B**). The zebrafish were administered with MVM425 via immersion challenge (2 × 10^7^ CFU/mL) (*n* = 30 fish per group). Statistical differences were evaluated by log-rank (Mantel–Cox) test, * *p* < 0.05.

We selected the recombinant *S. limacinum* SR21-FBA (SR21-gapdh-Gf-Gb) with the highest expression level of antigen protein for the immunization experiment. Based on the determination of the optimized proportion of *Schizochytrium* in the feed, the experimental group of zebrafish was fed a diet containing 15% recombinant SR21 expressing FBA (SR21-FBA group), while the others two groups were given either basal commercial feed (Control group) or a mixture of 15% WT *S. limacinum* (SR21 WT group). Two rounds of immunization were conducted to assess the immunization efficacy in zebrafish. The intestinal tissues of zebrafish were collected at 7, 14, and 28 days post-vaccination and the challenge experiment was performed on the 7th day after the completion of immunization (Figure 3A).

On the 7th day following a secondary immunization, zebrafish underwent an immersion challenge assay with *V. anguillarum* to evaluate the efficacy of the immunization in conferring cross-protection (Figure 3B). Notably, both the wild-type SR21 group and the SR21-FBA group demonstrated significant protective effects compared to the control group fed with commercial fish feed (*p* < 0.05). Among them, the protective effect of the SR21-FBA group was slightly superior to that of the wild-type SR21 group.

**Figure 4:**
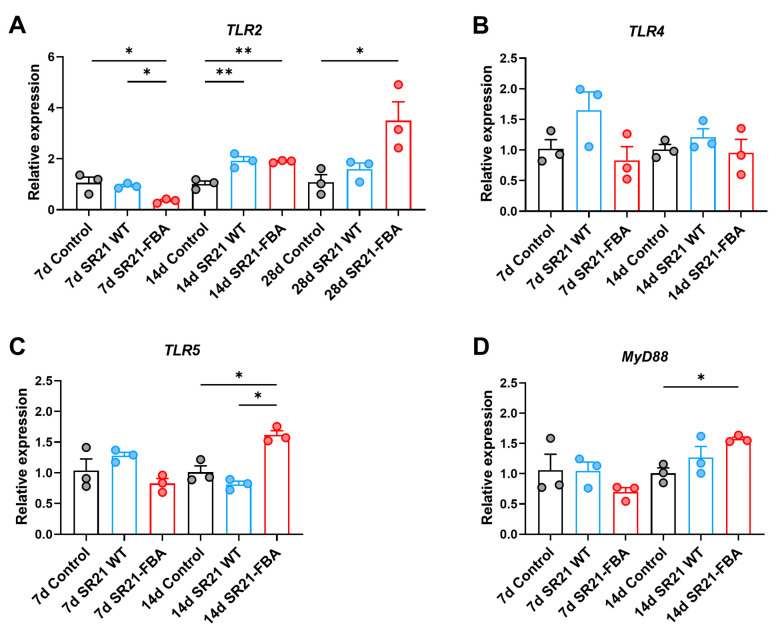
RT-qPCR analysis of genes involved in the toll-like receptor signaling pathway after secondary vaccination at different time points. Relative expression of *TLR2* (**A**), *TLR4* (**B**), *TLR5* (**C**), and *MyD88* (**D**) (*n* = 3 pools, 10 fish per pool). Data are presented as the means ± SD. * *p* < 0.05, ** *p* < 0.01.

To better evaluate vaccine efficacy, we examined the expression of key immune genes in zebrafish, including toll-like receptors, cytokines, and molecules related to antigen presentation. Following oral immunization with recombinant *S. limacinum* SR21-FBA in zebrafish, the gene expression of key immune signaling molecules was observed. These genes include *TLR2*, *TLR4*, *TLR5* (toll-like receptors 2, 4, and 5), and *MyD88* (myeloid differentiation primary response protein) (Figure 4A–D). The expression of these genes did not exhibit upregulation at the 7th day post-immunization. However, a notable shift occurred at the 14th day post-immunization, with a significant increased expression level of *TLR2* and *TLR5* (*p* < 0.01), as well as of *MyD88* (*p* < 0.05). This phenomenon revealed the dynamic response of the zebrafish immune system following vaccination, indicating that these immune signaling pathways were mildly activated during the initial phase and then entered a more active state.

**Figure 5:**
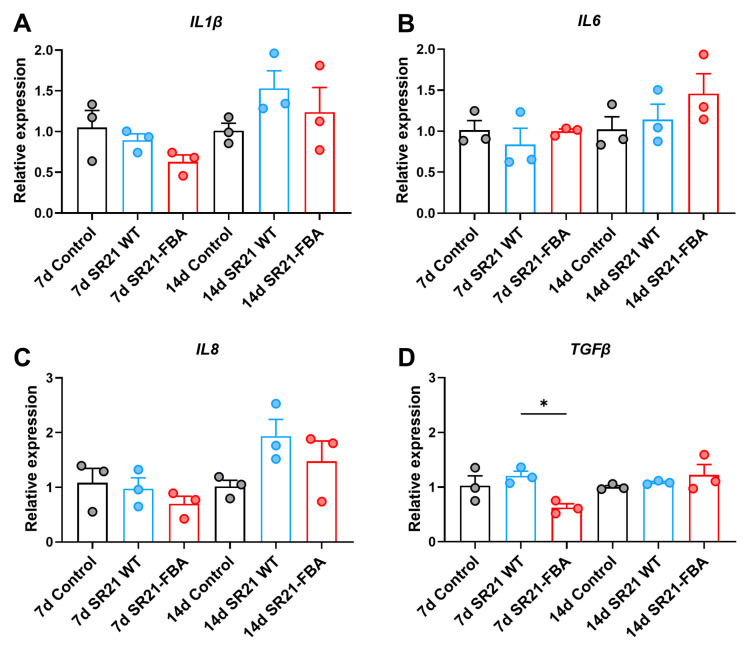
RT-qPCR analysis of cytokine genes after secondary vaccination in different time points. Relative expression of *IL1β* (**A**), *IL6* (**B**), *IL8* (**C**), and *TGF-β* (**D**) (*n* = 3 pools, 10 fish per pool). Data are presented as the means ± SD. * *p* < 0.05.

After oral immunization of zebrafish with recombinant SR21-FBA, it was noted that the expression of cytokine genes *IL1β*, *IL6*, *IL8*, and *TGFβ* did not show a significant upregulation at the 7th day post-immunization (Figure 5A–D). However, there was an increased expression trend for *IL1β*, *IL6*, and *IL8* at the 14th day post-immunization, although without statistical significance. These findings suggested that the immune response of zebrafish to the vaccination was gradual and moderated, with cytokine gene expression levels in the intestine exhibiting a tendency to rise but not yet reaching a level of notable difference.

**Figure 6:**
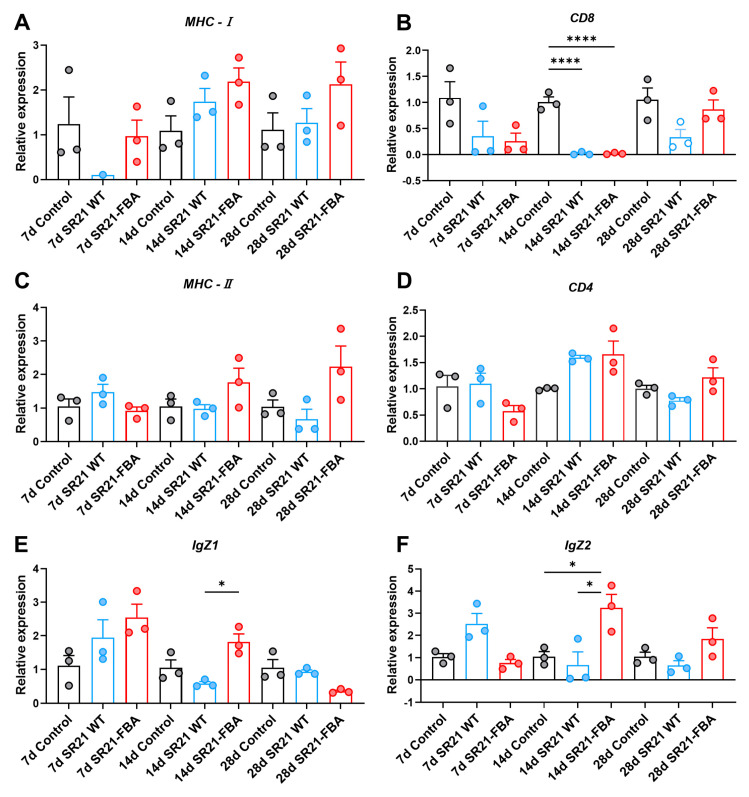
RT-qPCR analysis of genes related to antigen presenting and mucosal antibody after secondary vaccination at different time points. Relative expression of *MHC-I* (**A**), *CD8* (**B**), *MHC-II2* (**C**), *CD4* (**D**), *IgZ1* (**E**), and *IgZ2* (**F**) (*n* = 3 pools, 10 fish per pool). Data are presented as the means ± SD. * *p* < 0.05, **** *p* < 0.0001.

Then, a detailed examination of the gene expression of molecules associated with antigen presentation pathways was conducted at the 7th, 14th, and 28th days post-immunization. The results indicated that the oral SR21-FBA vaccine was capable of simultaneously activating both the major histocompatibility complex class I and II (MHC-I and MHC-II) antigen presentation pathways within zebrafish at the 14th and 28th day post-immunization. The expression of *MHC-I* was the highest among the three groups on the 14th and 28th days post-immunization (Figure 6A, not statistically significant). Similarly, the expression of *MHC-II* was also the highest on these days (Figure 6C, not statistically significant). However, the expression of *CD8* (cluster of differentiation 8) exhibited a downregulation trend in the SR21-FBA group (Figure 6B). In contrast to the expression of *CD8*, the expression of *CD4* exhibited an upregulation trend in this group both at the 14th and 28th day post-immunization (Figure 6D). Notably, as for the zebrafish-specific mucosal immune response, the expression of *IgZ1* showed significant upregulation within two weeks and expression of *IgZ2* presented a significant trend towards upregulation after two weeks (Figure 6E,F, *p* < 0.05). These findings elucidate the immune regulatory mechanisms in zebrafish following oral immunization, demonstrating that the vaccine effectively elicited a specific immune response in zebrafish. This provides valuable scientific insights for further research and development of novel vaccines.

**Figure 7:**
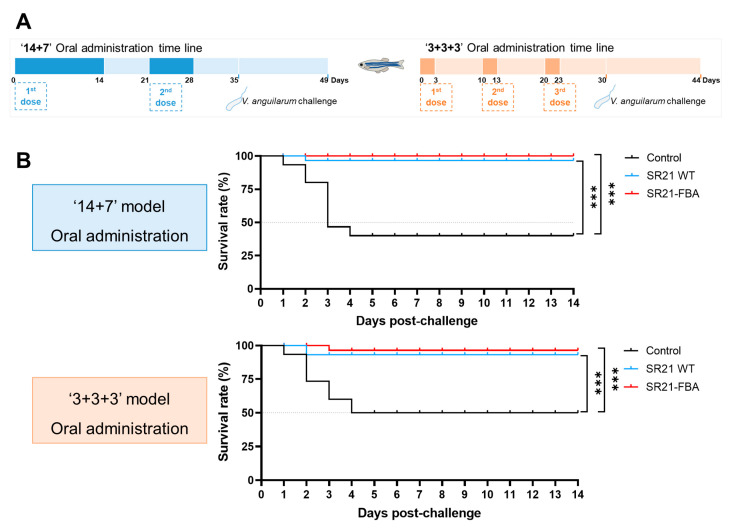
The protective effects of different immunization modes in zebrafish. Oral administration time line scheme (**A**) and survival curves of zebrafish challenged with *V. anguillarum* MVM425 (**B**). The fish were administrated with MVM425 via immersion challenge (3 × 10^6^ CFU/mL) (*n* = 30 fish per group). Statistical differences were evaluated by log-rank (Mantel–Cox) test, *** *p* < 0.001.

To better assess the protective effect against *V. anguillarum* MVM425, we conducted experiments in zebrafish using different oral administration models (14 + 7 model and 3 + 3 + 3 model, Figure 7A). The results demonstrated that there was no significant difference in the protective effects between the two models (Figure 7B).

### 2.4. Oral Administration with Edible Schizochytrium-Based Vaccine in Koi Carp

**Figure 8:**
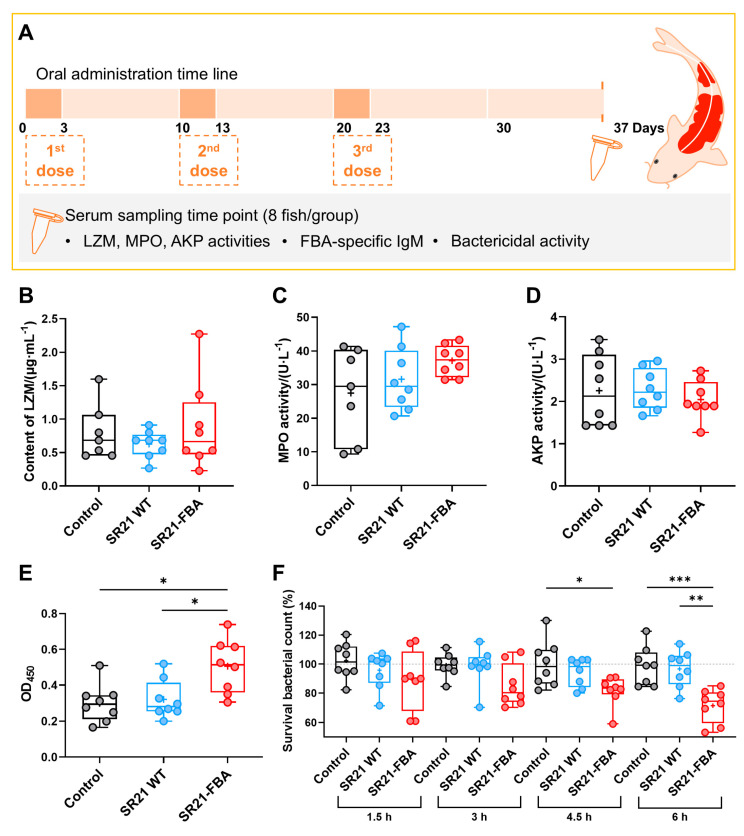
Three-dose immunization experiment in koi carp. Koi carp experimental scheme diagram (**A**). Serum enzyme activities of koi carp post-vaccination with recombinant vaccines, including LZM (**B**), MPO (**C**), and AKP (**D**) activities (*n* = 8 fish per group). The levels of serum IgM antibody (**E**) directed against antigen protein FBA post-vaccination (*n* = 8 fish per group). The serum bactericidal activity (**F**) against *E. tarda* EIB202 at different incubation time points (*n* = 8 fish per group). All individual data and the mean (+) are represented. Statistical comparisons were compared by one-way ANOVA, * *p* < 0.05, ** *p* < 0.01, *** *p* < 0.001.

Taking into account the economic viability of feed preparation, we conducted an immunization efficacy assessment in ornamental koi fish with three rounds of immunization (3 + 3 + 3 model). Sera samples were collected on the 14th day after the completion of immunization (Figure 8A). During the feeding period, we weighed the koi fish from each group and no significant differences were observed. As in the results of immunization experiments in zebrafish, both SR21 WT and SR21-FBA exhibited immunoprotective effects. To better estimate the efficacy of the oral vaccine SR21-FBA, we assessed the changes in the non-specific and specific immune parameters in the sera from koi carp.

To assess the non-specific immune status, we measured the activities of lysozyme (LZM), myeloperoxidase (MPO), and alkaline phosphatase (AKP) in the sera of koi fish on the 14th day post-oral immunization (Figure 8B–D). The results indicated that there were no significant differences in LZM activities among the three groups. The AKP activity in the SR21-FBA-immunized group showed a slight trend of decrease. However, regarding MPO activity, there was an upward trend in the immunized group compared to the control group, although it did not fully reach statistical significance. This might suggest that the immune system might have activated the non-specific immune defense mechanisms of the koi fish.

To analyze the levels of FBA-specific IgM antibodies in the sera against the antigen protein FBA of pathogen EIB202, we conducted an ELISA test on the sera of koi fish 14 days post-oral immunization, using purified FBA as the antigen (Figure 8E). The SR21-FBA oral vaccine group exhibited a significant increase in FBA-specific antibody levels in the sera post-immunization compared to both the control group (*p* < 0.05) and the SR21 WT group (*p* < 0.05). This finding indicates that the SR21-FBA oral vaccine effectively induces a specific immune response against the FBA antigen in koi fish, which may enhance their immune protection against pathogens.

To assess the bactericidal capacity of the antisera, the sera from koi fish at the 14th day post-oral immunization was incubated with the pathogen EIB202 (Figure 8F). No significant difference was observed between the SR21 WT group and the control group. After 1.5 h of incubation, the SR21-FBA oral vaccine group exhibited a notable trend towards increased bactericidal activity against the pathogen compared to the other two groups. Notably, the SR21-FBA group demonstrated a significant bactericidal effect against the pathogen EIB202 at 4.5 h (*p* < 0.05, compared to the control group) and at 6 h (*p* < 0.001, compared to the control group). After 6 h post-incubation, there was a marked difference in bactericidal efficacy between the oral vaccine SR21-FBA group and the wild-type (SR21 WT) fed group (*p* < 0.01). These results suggest that the sera of koi fish immunized with SR21-FBA have enhanced bactericidal activity, which may be related to the activation of specific immune defenses associated with antigen proteins.

## 3. Discussion

Vaccination is an effective preventive strategy for disease control and can elicit long-lasting specific immunity in aquatic animals. Currently, numerous studies are developing various types of vaccines, including injectable, immersion, oral, and spray formulations [23]. Although injectable vaccines are currently more effective, they also have limitations due to the potential burden on the fish and the risk of wound infection [24]. Developing efficient immunization methods that circumvent these drawbacks is a top priority in current research. Oral vaccines can avoid these shortcomings. Current oral vaccines utilize biocompatible materials for antigen encapsulation, including biopolymers (e.g., alginate), microorganisms (e.g., *Lactococcus*, *Lactobacillus*, *Bacillus subtilis*, yeast), plant materials (e.g., *Nicotiana benthamiana*), and microalgae (e.g., *Chlamydomonas reinhardtii*) [25,26,27]. These studies provide valuable information for the development of future edible vaccines, by ensuring the stability of antigens in the harsh gastrointestinal environment and their uptake in the stomachs of some fish.

In this study, we successfully expressed the antigenic protein FBA in the novel edible chassis cell *S. limacinum* SR21. Then, the short-term immunity effects of recombinant SR21 were evaluated in zebrafish and koi carp via oral administration. Although the results showed that it induced changes in the expression of immune related genes and play a certain immunoprotective role, this study offers new insights into the research of oral vaccines for aquaculture. Future research will be gradually optimized based on the following discussions.

It is noted that the 2A peptide from porcine teschovirus did not fully exhibit cleavage efficiency in our test. This short ‘self-cleaving’ peptide (usually 16–20 amino acids) is derived from picornavirus. It works by enabling the ribosome to skip the synthesis of the glycyl–prolyl peptide bond at the C-terminus of the 2A element, leading to the separation of the 2A sequence terminus from the downstream product [28]. We did not access the efficacy of other types of 2A peptides, as it did not seem essential for achieving high expression of heterologous proteins. Nevertheless, it is an advantageous element to facilitate the co-expression multiple genes.

We aim to obtain *Schizochytrium* transformants with high expression levels of antigen proteins. In the complex environment of the gastrointestinal tract, the cell wall and membrane structures of *Schizochytrium* cells facilitate the slow release and degradation of their intracellular substances. We believe that the accumulation of a higher quantity of antigen proteins within *Schizochytrium* cells is positively correlated with the likelihood of their successful delivery to the gastrointestinal tract of fish. The antigen protein might be recognized by the gut-associated mucosal immune system, thereby stimulating a series of local and systemic immune responses. However, there are still some methodological challenges that need to be urgently addressed [11]. The genetic manipulation of *Schizochytrium* needs to be continuously optimized. Recent reports have indicated that several recombinant bioactive proteins have been successfully expressed utilizing *Schizochytrium* sp. ATCC20888 as a host. These include the GP1 protein of *Zaire ebolavirus* (1.25 mg/g fresh weight), the B subunit of the heat-labile enterotoxin from *E. coli* (LTB, 0.12 mg/g fresh weight), hemagglutinins of the influenza H1N1 and H5N1 viruses (5–20 mg/L), tumor-associated antigens (TAAs) for breast cancer (637 μg/g fresh weight), and the chimeric protein Tc24:Co1, which combines the 24 kDa flagellar calcium-binding protein (Tc24) of parasite *Trypanosoma cruzi* with the Co1 M cell-targeting ligand (300 μg/g dry biomass) [29,30,31,32,33]. In this study, we successfully used *S. limacinum* SR21 as chassis to achieve the expression of the antigenic protein FBA. The expression of the antigen protein in the recombinant strain SR21-gapdh-Gf-Gb was quantified to be approximately 200 μg/g dry biomass. Moreover, the protein FBA expression of the transformants was stable with prolonged culture time. In further studies, we plan to enhance the expression of antigen proteins and explore their precise localization within the cell.

It is well known that the intestine plays a crucial role in the mucosal immune system. The more that antigen proteins are expressed and accumulated in *Schizochytrium* cells, the fewer immune tolerance events occur as a result of repeated stimulation with low doses of antigen [34]. The cell size ranges from 2 to 30 μm in diameter for zoospores and is larger than 30 μm for mature zoosporangia. The cell size of *Chlamydomonas* is similar to that of *Schizochytrium*. The fluorescent signal was successfully captured within the intestinal mucosal layer of zebrafish after oral administration with GFP-expressing *Chlamydomonas reinhardtii* [35]. *Schizochytrium* sp. has been used to produce a novel zika virus vaccine candidate; mice were orally immunized with intact *Schizochytrium* cells (expressing 18 μg ZK antigen), which induced the production of serum-specific IgG and intestinal IgA [31]. In another study, oral administration of a whole-cell *Schizochytrium* sp. vaccine (expressing 7.5 or 15 μg of chemic protein Tc24:Co1) triggered the production of specific anti-Tc24 antibody IgG in sera and IgA in intestinal mucus [32]. All of these studies demonstrate that microorganisms of this size can be used as carriers for oral vaccines.

In our previous studies, injectable immunization was typically administered to fish with purified antigen proteins accompanied by adjuvants. The dosage of antigen proteins injected intramuscularly was 1.5 μg in zebrafish (~0.2 g weight) and intraperitoneally was 35 μg in turbot (~30 g weight) [21,22]. In this study, we designed different immunization protocols for zebrafish and koi, respectively. To prevent the degradation of the antigen proteins, we collected recombinant transformants by cryogenic freeze-drying. The total amount of antigen protein ingested by the zebrafish was calculated to be at least 20 μg/fish, while the total amount ingested by the koi was theoretically at least 500 μg/fish.

Considering the small size of zebrafish and the potential stress associated with injection, we assessed the cross-protection effect of recombinant *Schizochytrium* against bacterial pathogen MVM425 through immersion challenges. Additionally, we evaluated the expression of gut-related immune genes for a comprehensive assessment of recombinant *Schizochytrium*. For the larger ornamental koi carp, we conducted challenge experiments via injection and evaluated several serological immunity indicators.

The survival curves demonstrate that both wild-type and recombinant *Schizochytrium* feedings provided immunoprotection to both fish species under investigation. However, there was no significant difference between the recombinant and wild-type SR21, as expected. This was similar to a study by Shi et al., where a diet containing 12% *Schizochytrium* sp. provided some protection to zebrafish from *Edwardsiella piscicida* infection [36]. The bioactive compounds in *Schizochytrium* are closely linked to immune stimulation in aquatic animals and the inclusion of whole-cell *Schizochytrium* in fish feeds has been shown to improve immune responses and enhance survival under stressful conditions and infectious challenges [37].

However, despite the small difference of the survival curves between the SR21 WT and SR21 FBA groups mentioned above, the SR21-FBA group stimulated specific immune responses in the zebrafish gut, as indicated by the quantitative expression analysis of gut-related immune genes after immunization. On the 14th day after immunization, the expression of *TLR2*, *TLR5*, and *MyD88* was significantly upregulated in the SR21-FBA group compared to the other groups. Correspondingly, the expression of *IL1β*, *IL6*, *IL8*, and *TGFβ* in this group also tended to be up-regulated at this time point. The expression of *MHC-I* and *MHC-II* in the SR21-FBA group was also up-regulated compared to the other control groups on both 14th and 28th day post-immunization, but it was not significant enough. Notably, the gene expression of *CD4* in this group showed the same trend at both time points. Regarding the antibodies related to mucosal immunity, the expression of *IgZ1* and *IgZ2* was significantly up-regulated at the time point of 14th day post-immunization compared to other groups. These changes implied that our recombinant *Schizochytrium* successfully stimulated the immune response in the zebrafish intestine.

To better assess whether our constructed vaccine strain could stimulate a global humoral immune response in fish through oral administration, we evaluated the efficiency of the recombinant vaccine in ornamental koi fish. Both non-specific and specific immunological indicators in sera were analyzed. Among the non-specific immune indicators, such as lysozyme, myeloperoxidase, and alkaline phosphatase activities, LZM, a non-specific protein-based defense factor, plays an important role in aquatic animal resistance to pathogen infection [38]. AKP is an important hydrolyzing enzyme with various physiological functions, while MPO, released predominantly by neutrophils, is a key component of the innate immune system against pathogen invasion [39]. Serological analysis of koi showed no significant changes in the above non-specific immunological indicators among the groups, whereas MPO activity exhibited an upward trend in sera from the immunized group (SR21-FBA). Similar to our experiment, Sun et al. consistently fed a diet containing 1% *Schizochytrium* to humpback groupers (*Cromileptes altivelis*), finding no statistically significant difference in sera LZM activity compared to the control diet group over a 4-week feeding period. However, sera AKP activity was significantly increased in the fish fed diets supplemented with *Schizochytrium* compared to the controls at 2 and 4 weeks, while there were no significant differences at other time points [40]. Yao et al. reported that replacing dietary fish oil (75 g/kg dry biomass) with *Schizochytrium* algal oil significantly increased AKP activity in the intestinal tract and brush border membranes of greater yellow croaker juveniles [41]. Conversely, adding 15% *Schizochytrium* to the diet reduced AKP activity in the intestine and brush border membrane of turbot (*Scophthalmus maximus*) larvae [42]. Notably, in our study, the sera MPO activity in koi fed recombinant *Schizochytrium* showed an upward trend compared to the other two groups. This increase may be attribute to the recognition of the recombinant antigen protein by neutrophils, leading to increased release of MPO.

Humoral immunization is usually assessed by specific antibody concentrations [43]. Our study on koi carp collected the sera samples at the 14th day after immunization. Although we did not monitor changes in serum antibody levels throughout the longer experimental period, our study showed that specific IgM antibody levels in the sera were significantly higher in koi fed recombinant *Schizochytrium* diets compared to the other two control groups. Interestingly, the trend of serum bactericidal activity assay aligned with the serum antibody levels, with koi fed with recombinant *Schizochytrium* SR21-FBA showing a significant bactericidal effect against the pathogen EIB202, whereas the sera of koi fed with SR21 WT did not exhibit a significant specific bactericidal effect.

This is the first study to investigate the immunization efficacy of a *Schizochytrium*-based oral vaccine in fish, as far as we know. Our results indicate that designing oral vaccines with *Schizochytrium* is feasible and demonstrates a certain level of immune efficacy. We confirmed that the oral administration of recombinant *Schizochytrium* effectively elicited an immune response in koi carp through assays of MPO activity, specific antibodies concentrations in the sera, and bactericidal effects. In further research, the long-term immune effects triggered by the oral immunization of our vaccine will be analyzed. Additionally, this study is a preliminary study and there is a need to prove the safety of using of this transgenic microbe in open nature.

## 4. Materials and Methods

### 4.1. Strains

*Escherichia coli* DH5α (stored in our laboratory) was used for the cloning and replicating of the constructed plasmid. *S. limacinum* SR21 was obtained from Ningbo Futian Biotech Co., Ltd (Ningbo, China). The pathogen strains *Vibro anguillarum* MVM425 and *E. tarda* EIB202 were preserved in our laboratory [44].

### 4.2. Construction of Plasmid

The homologous upstream and downstream arm sequences from the SR21 genome (https://mycocosm.jgi.doe.gov/Aurli1/Aurli1.home.html) (accessed on 11 December 2024) were amplified using PrimeSTAR Max DNA Polymerase (Takara Biotechnology, Beijing, China). The endogenous promoter and terminator sequences from SR21 genome were amplified to drive the expression of antigen gene *fba*. The sequence of the antigen gene *fba* (Accession number: ACY85789.1) was amplified from the EIB202 genome (CP001135.1). The marker gene *Ble* was synthesized by Tsingke (Beijing, China). All sequences were cloned into a T-vector (Takara Biotechnology, China) using 2 × MultiF Seamless Assembly Mix (ABclonal Technology, Wuhan, China) according to the instructions. All primers (Supplementary Data Table S1) used for construction were synthesized by Tsingke (Beijing, China). The DNA sequences of genes of interest were all confirmed by DNA sequencing by Tsingke (Beijing, China). The plasmid was linearized by *Not*Ⅰ and *Bst*BⅠ (Thermo Fisher Scientific, Waltham, MA, USA) for later transformation.

### 4.3. Transformation of Schizochytrium

The strain SR21 was cultured in seed medium (30 g/L glucose, 10 g/L yeast extract, 12 g/L Na_2_SO_4_, 2 g/L MgSO_4_, 1 g/L KH_2_PO_4_, 1 g/L (NH_4_)_2_SO_4_, 0.65 g/L K_2_SO_4_, 0.5 g/L KCl, and 0.13 g/L CaCl_2_, pH 6.5). The electro-transformation followed the electroporation protocol of *Aurantiochytrium limacinum* [45]. Briefly, cells in the logarithmic phase were harvested at 5000× *g* and washed once with BSS (10 mM KCl, 10 mM NaCl and 3 mM CaCl_2_) buffer and twice with 50 mM sucrose solution. The cells were then resuspended in 50 mM sucrose to a final concentration of 2 × 10^8^ cells/mL. Then, 200 μL of the cell resuspension was mixed with 5 μL of linearized plasmid DNA (Figure S1, pGb2Af or pGf-Gb, approximately 5 μg) and transferred to a chilled 2 mm-gap electroporation cuvette (Bio-Rad, Beijing, China). The dry cuvette was placed in the electroporation chamber and subjected to a pulse using the Gene Pulser Xcell Electroporation System (Bio-Rad, Hercules, CA, USA) at a voltage of 1.5 kV. After pulsing, the cells were immediately transferred to 1 mL seed medium (with 50 mM sucrose) and incubated at 28 °C overnight. The cells were centrifuged at 3500× *g* and spread onto agar plates (with 100 μg/mL zeocin (Thermo Fisher Scientific, USA)). After plating on selective medium colonies generally became visible after 3–5 days of incubation at 28 °C. The linearized plasmid was stably integrated in the genome of *Schizochytrium* by homologous recombination. The integration of genomes was verified via colony PCR (Supplementary Data Figure S2), followed by DNA sequencing of the PCR products amplified using primers flanking the modified regions of the SR21 genome. All primers used for verification of transformants (Supplementary Data Table S1) were synthesized by Tsingke (Beijing, China). The sequencing was performed by Tsingke (Beijing, China).

### 4.4. Western Blot

For protein expression analysis, the recombinant strain SR21-gapdh-Gf-Gb was cultured overnight in the seed medium at 28 °C with shaking. The cells were harvested and lyophilized overnight. Protein concentrations were determined via BCA assay (Yeasen, China) following the protocol. Then, 10 mg powder was boiled in the 5 × SDS-PAGE protein loading buffer (Yeasen, Shanghai, China) and loaded into 12% polyacrylamide gel under denaturing conditions. After that, proteins were transferred onto a polyvinylidene fluoride (PVDF) membrane (Cytiva, Marlborough, MA, USA) and probed with mouse anti-FBA antibody (1:10,000, customized from GL Biochem, Shanghai, China), then with horseradish peroxidase (HRP)-conjugated affinipure goat anti-mouse IgG (H + L) (1:10,000, Proteintech, Rosemont, IL, USA) as a secondary antibody. The protein bands were visualized with a chemiluminescent substrate kit (Beyotime, Haimen, China).

### 4.5. Experimental Fish and Oral Vaccination Regime

Adult zebrafish (with an average weight of approximately 0.21 g per tail) were purchased from a local supplier in Shanghai, China. The koi carp (with an average weight of approximately 60 g per tail) were supplied by Ningbo Sansheng Biological Technology Co., Ltd. (Ningbo, China). Before initiating the experimental procedures, all fish were pre-cultured for two weeks in a breeding system. The water temperature was maintained at 25–26 °C. Feed preparation was followed our previous study [10]. The treatment groups were as follows: (1) Control groups, fed with commercial feeds; (2) 7.5, 15, and 30% SR21, fed a diet containing 7.5, 15, and 30% (*w*/*w*) wild-type (WT) SR21 powder, respectively; (3) SR21 WT, fed a diet containing 15% WT SR21; and (4) SR21-FBA, fed a diet containing 15% recombinant SR21 expressing FBA. The zebrafish were fed twice a day and koi carps were fed three times a day.

Some of the zebrafish were orally administered with two rounds of immunization (Figure 3 and Figure 7). The first immunization involved a continuous 14-day feeding period with recombinant *Schizochytrium* feed (SR21-FBA group), followed by a 7-day interval during which a commercial diet was provided. Then a second immunization phase with another 7-day regimen of recombinant feed. The other zebrafish and koi carp were subjected to a three-stage immunization protocol (Figure 7 and Figure 8), with each stage consisting of a 3-day continuous feeding period of recombinant feed, separated by 7-day intervals of commercial feed. Fish fed with commercial feed served as the blank control (control group), while fish fed with feed supplemented with wild-type *S. limacinum* SR21 served as the negative control (SR21 WT group). The immune experimental feeds were formulated by incorporating 15% (*w*/*w*) lyophilized SR21 powder. All fish were distributed in three treatment groups in triplicate tanks.

### 4.6. Sample Collection

The intestinal tissues (midgut and hindgut) of the zebrafish were harvested at 7-, 14-, and 28-days post-vaccination (each pool comprising 10 individuals and three replicate pools per time point from each group). The samples were submerged in RNA store reagent (Tiangen Biotech, Beijing, China) overnight prior to being stored at −85 °C for subsequent RNA extraction.

For the koi carp, a short-term time point of 14 days post-immunization was selected for blood collection from each group (8 fish per group). Following a period of sedimentation at 4 °C overnight, the samples were centrifuged at 1000× *g* for 10 min to separate the sera. The sera were then carefully transferred to centrifuge tubes and cryopreserved at −85 °C.

### 4.7. Challenge Experiment

Zebrafish (30 individuals per group) were subjected to a 10 min bath challenge with an aerated bacterial physiological seawater suspension containing *V. anguillarum* MVM425. Following the challenge, the fish were transferred to clean water for a 5 min recovery period before being returned to their rearing environment. All of the challenge doses were determined by preliminary experiments. Then, the fish were carefully monitored and returned to a controlled rearing environment, where their health and behavioral responses were closely observed and documented.

### 4.8. RNA Extraction and Real-Time Quantitative Polymerase Chain Reaction (RT-qPCR)

All total RNA was extracted using TRIzol (Thermo Fisher Scientific, USA) according to the manufacturer’s protocol. The concentration of RNA was determined by NanoDrop One^C^ (Thermo Fisher Scientific, USA). The cDNA was synthesized utilizing a PrimeScript RT reagent kit (Perfect Real Time) (Takara Biotechnology, China). qRT-PCR was performed by an Applied Biosystems 7500 Fast Real-Time PCR System (Thermo Fisher Scientific, USA) using Hieff UNICON universal blue qPCR SYBR green master mix (Yeasen, China). The results were quantified by the 2^−ΔΔCt^ method [46] with *β-actin* as the reference gene. Primers for RT-qPCR are listed in Supplementary Data Table S2.

### 4.9. Detection of Serum Non-Specific Immune Parameters

The non-specific immune enzymatic activities in the sera, including lysozyme (LZM), myeloperoxidase (MPO), and alkaline phosphatase (AKP), were assessed. A lysozyme assay kit (A050), myeloperoxidase assay kit (A044), and alkaline phosphatase assay kit (A059) were purchased from Nanjing Jiancheng Bioengineering Institute (Nanjing, China) and utilized to determine the activity these enzymes according to the manufacturer’s instructions, respectively.

### 4.10. Detection of Antigen Protein FBA-Specific IgM

The levels of sera antibodies directed against the antigen protein FBA were assessed with an enzyme-linked immunosorbent assay (ELISA). The ELISA plates (NEST Biotech, China) were coated with 100 μL purified antigen protein FBA, which was dissolved at a concentration of 20 μg/mL in carbonate bicarbonate buffer (1.59 g/L Na_2_CO_3_, 2.93 g NaHCO_3_, pH 9.6) and incubated overnight at 4 °C. The expression and purification of the antigen protein FBA from the pathogen EIB202 adhered to the previous method [22]. Plates were then blocked with 200 μL PBS containing 0.05% Tween (*v*/*v*) and 1% bovine serum albumin (BSA) (*w*/*v*) at 22 °C for 2 h. Then, 100 μL sera samples (diluted at 1:10 in PBST) were added to each well and incubated at 22 °C for 3 h, after which a mouse anti koi carp IgM antibody (Aquatic Dignostics, Oban, SCO, UK) was added to the plates (100 μL per well at 1:33 in PBST) at 22 °C for 1 h. The second antibody, HRP-conjugated affinipure goat anti-mouse IgG (H + L) (Proteintech, USA) (100 μL diluted at 1:10,000 in PBST), was subsequently added to each well at 22 °C for 1 h. Tetramethylbenzidine (TMB) chromogen was added to the plates (100 μL per well) and incubated at 37 °C for 0.5 h. The reaction was stopped by adding 50 μL stop solution (2 M H_2_SO_4_). The optical density (OD) was measured with microplate reader (BioTek 800TS, Winooski, VT, USA) at 450 nm. The plate was washed five times with 200 μL PBST between each of the two steps. All samples were run in duplicate.

### 4.11. Detection of Serum Bactericidal Activity

*E. tarda* EIB202 was cultured in LB medium at 28 °C overnight to achieve a concentration of 10^4^ CFU/mL. Subsequently, 50 μL of the bacterial suspension was harvested and mixed with 50 μL sera and incubated at 28 °C. Thereafter, 10 μL aliquot of the mixed sample was taken every 1.5 h for the drop plate assay. Non-immunized sera from commercially fed fish served as a control. The bacterial survival count (%) was determined by calculating the ratio of the colony counts on the experimental plates to the mean colony count on the control plates.

### 4.12. Statistical Analysis

All analyses were performed with GraphPad Prism v9.0 (GraphPad software, Boston, MA, USA). The normality (Shapiro–Wilk test) was checked for all data. To assess statistical differences among dietary treatments at each time point, data (mRNA expression, LZM, MPO, AKP activities, and sera antibody levels) were statistically analyzed by one-way analysis of variance (ANOVA) followed by Holm–Šídák test, with diet as the explanatory variable. The survival rate data were compared using the Kaplan–Meier method, further evaluated by log-rank (Mantel–Cox) test. * *p* < 0.05, ** *p* < 0.01, *** *p* < 0.001, **** *p* < 0.0001.

## 5. Conclusions

In summary, we determined the optimal proportion of *S. limacinum* SR21 in fish feed. Then, we constructed stable *Schizochytrium* transformants expressing the antigen protein FBA and developed them as novel oral vaccine for the aquaculture industry. Zebrafish and koi carp were utilized to evaluate the oral immunization efficacy of the *Schizochytrium* transformants. The results demonstrated that the oral administration of recombinant *Schizochytrium* led to alterations in the expression of immune-related genes and conferred some degree of immunoprotection in fish. As for zebrafish, the expression levels of *MHC-I* and *MHC-II*, which are involved in cell-mediated adaptive immune responses, as well as the highly specialized antibody gene *IgZ*, which is crucial for mucosal immunity, were significantly upregulated on the 14th and 28th days post-immunization. In koi carp, specific antibodies against the antigenic protein FBA were successfully triggered in the sera and the immune sera exhibited significant bactericidal activity against *E. tarda* EIB202.

## Figures and Tables

**Table 1:** The weight and survival rate of zebrafish fed with different concentrations of SR21.

Parameters/Groups	Control	7.5% SR21	15% SR21	30% SR21
IW (g)	0.317 ± 0.003	0.315 ± 0.004	0.314 ± 0.005	0.317 ± 0.001
FW (g)	0.346 ± 0.003	0.363 ± 0.011	0.387 ± 0.006	0.363 ± 0.006
SR (%)	100	100	100	100

Note: IW, Initial weight; FW, Final weight; SR, Survival rate.

## Data Availability

The data presented in this study are available on request from the corresponding author.

## Supplementary Materials

The following supporting information can be downloaded at: https://www.mdpi.com/article/10.3390/md22120555/s1, Table S1: The primers used for construction of recombinants; Table S2: The primers used for RT-qPCR; Figure S1: The scheme of integration via homologous recombination; Figure S2: Identification of Gf-Gb transformants via colony PCR.
