# Supplementary material for: Transgenic Schizochytrium as a Promising Oral Vaccine Carrier: Potential Application in the Aquaculture Industry
Source: Mar Drugs. 2024 Dec 12;22(12):555. doi: 10.3390/md22120555 (PMC11678113; doi:10.3390/md22120555)
Supplement: Supplementary file 1 [file marinedrugs-22-00555-s001.zip › marinedrugs-3341262-supplementary.pdf]

Supplementary materials, *Marine drugs*

Transgenic *Schizochytrium* as a Promising Oral Vaccine Carrier: Potential Application in the Aquaculture Industry

Ke Ma[1,2], Lei Deng[1,2], Yuanjie Wu[3], Yuan Gao[3], Jianhua Fan[1,2,*], Haizhen Wu[1,2,*]

[1] State Key Laboratory of Bioreactor Engineering, East China University of Science and Technology, Shanghai 200237, P.R. China

[2] Department of Applied Biology, East China University of Science and Technology, Shanghai 200237, P.R. China

[3] Biopharmaceuticals R&D Department, Ningbo Sansheng Biological Technology Co., Ltd., Ningbo, Zhejiang Province 315012, P.R. China

*Corresponding author: Jianhua Fan, Haizhen Wu

Address: 130 Meilong Road, Shanghai 200237, P.R. China

Tel: +86-21-64252507; fax: +86-21-64252515;

Email: jhfan@ecust.edu.cn; wuhzh@ecust.edu.cn

Content

Figure S1. The scheme of integration via homologous recombination.

Figure S2. Identification of Gf-Gb transformants via colony PCR.

Table S1. The primers used for construction of recombinants.

Table S2. The primers used for RT-qPCR.

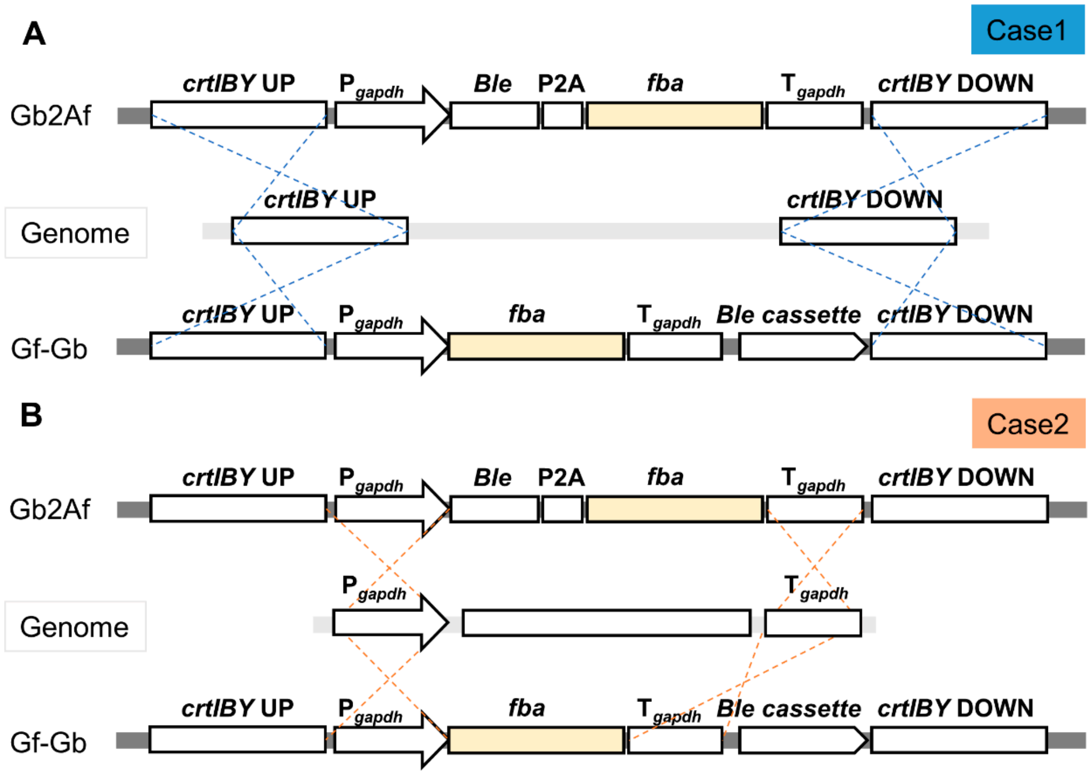

Figure S1. The scheme of integration via homologous recombination. Case 1_integration at the *crtIBY* gene locus (A), Case 2_integration at the *gapdh* gene locus (B). G2Af, the GAPDH promoter drive the co-expression of the marker gene *Ble* and the antigen gene *fba* (linked by a 2A sequence). Gf-Gb, the GAPDH promoter solely to drive the expression of the antigen gene *fba*.

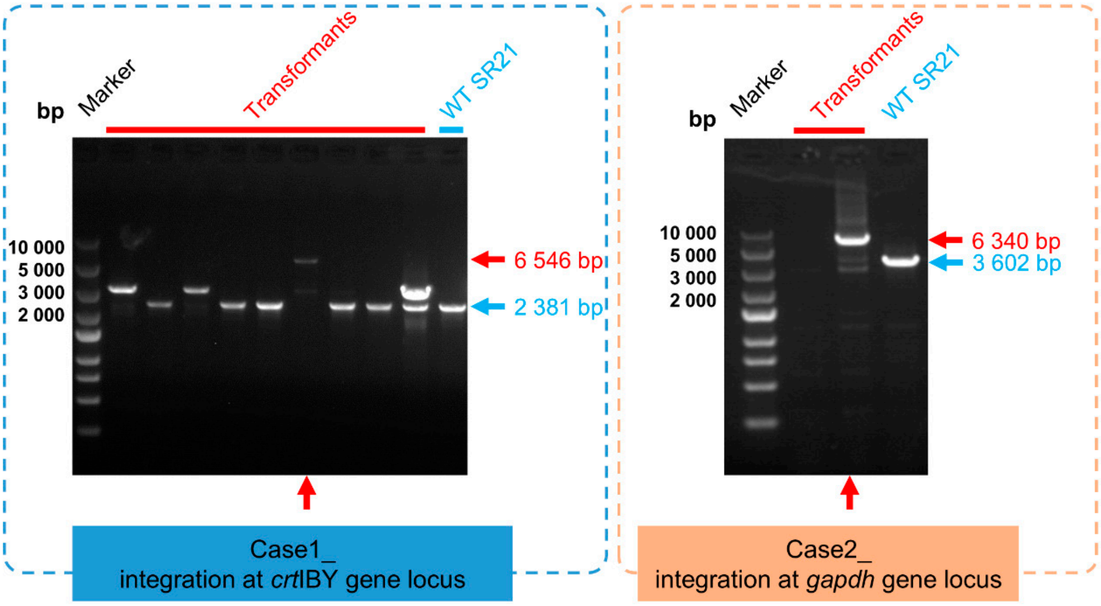

Figure S2. Identification of Gf-Gb transformants via colony PCR. The PCR products were amplified using primers flanking the modified regions of the SR21 genome. Red arrows indicate the positive transformants, blue arrows indicate the WT SR21. The DNA sequencing of the PCR products was following performed to confirm the integration.

Table S1

The primers used for construction of recombinants.

Primer	Sequence (5'-3')	Application
150841-U-F	AAAACGACGGCCAGTGAATTCGAGCTCG CCACCCAGGATGTCTGTC	Amplification of crtIBY upstream homologous sequence
150841-U-R	TGGAGCCCTCACAAGATCAAGGTACCGC TTAACCCTCCACTAGGG	
150841-D-F	CTCTTCTAGTGCTTCAAGGGATCCGATTT TTCCGCCGAGCTG	Amplification of crtIBY downstream homologous sequence
150841-D-R	GACCATGATTACGCCAAGCTTGCTCCAG AGTTCTCGAGTGC	
GAPDHp-F	TAGTGGAGGGTTAAGCGGTACCTTGATCT TGTGAGGGCTCCA	Amplification of endogenous promoter sequence
fba-GAPDHp-R	GTCAAAGATTTTAGACATTTTGCTTGGTG TTTATGT	
fba-GAPDHt-F	CACCACCACCACCACCACTAACCATGGA TGTACCCAATACCACACCGGTAGC	Amplification of endogenous terminator sequence
GAPDHt-R	CAGCTCGGCGGAAAAATCGGATCCCTTG AAGCACTAGAAGAG	
Gp-fba-F	GTCAAAGATTTTAGACATTTTGCTTGGTG TTTATGT	Amplification of antigen gene *fba* sequence
Gt-fba-R	CCATGGTTAGTGGTGGTGGTGGTGGTGC	(Separate cassette or linked

	AGCACGTCGATGCAGTT	by P2A sequence)
P2A-fba-F	AGCAAGCAGGAGACGTGGAAGAAAACCCCGGTCCTGTCGACATGTCTAAAATCTTTGAC	
P2A-Ble-R	TCCACGTCTCCTGCTTGCTTGAGCAGAGAGAAGTTCGTGGCGTCCTGCTCCTCGGCCAC	Amplification of marker gene *Ble* sequence
Ble-F	ACATAAACACCAAGCAAAATGGCCAAGTTGACCAGT	
crt-V-F	ATGGACGAAGCTAAAGAAAA	Colony PCR identification of SR21transformants at *crtIBY* gene locus
crt-V-R	ACCGTACTATAAACGAGGTA	
gapdh-V-F	CCGGACTGCCATTTGAACGA	Colony PCR identification of SR21transformants at *gapdh* gene locus
gapdh-V-R	GGCTCGGATTCGAATGAACG	

Table S2

The primers used for RT-qPCR.

Primer	Sequence (5’-3’)	Gene accession number
TLR2-F	TCTCCGTCTTGGTTTCAC	EU643837.1
TLR2-R	GGTCCCACAGTTGAGTATG	
TLR4-F	GGAATAATGGGCAGCCGTAAG	NM_212813.2
TLR4-R	AGCGACACCAGGAACTATCAATG	
TLR5-F	GAAACATTCACCCTGGCACA	NM_001130595.2
TLR5-R	CTACAACCAGCACCACCAGAATG	
MyD88-F	AACAACTTCGCTGGATAA	NM_212814.2
MyD88-R	GTTACTGGAATCGCCTCA	
IL6-F	TCAACTTCTCCAGCGTGATG	NM_001261449.1
IL6-R	TCTTTCCCTCTTTTCCTCCTG	
IL1β-F	TGGACTTCGCAGCACAAAATG	NM_212844.2
IL1β-R	GTTCACTTCACGCTCTTGGATG	
IL8-F	GTCGCTGCATTGAAACAGAA	XM_009306855.3
IL8-R	CTTAACCCATGGAGCAGAGG	
TGF-β-F	TGGGCTGGCGGTGGAT	NM_194386.2
TGF-β-R	CCTCTGGGTTCAGCGTGTT	
MHC1-F	GGAGTTCACCTTGCTTATGC	NM_194403.1
MHC1-R	CCCTCTGACCCATTCTTGT	
CD8-F	AAGAGCATAGCACCGTAG	NM_001040049.1

CD8-R	GACTTCCGTCTGCTTTGCG	
MHC2-F	TGACTCAACTGTCCGTGATA	NM_001005943.2
MHC2-R	CCATTAGCCATCTCCATAGTG	
CD4-F	GTGGTCTTCATCTTGCTTGT	NM_001135096.1
CD4-R	AATCCCTTTGGCTGTTTGTT	
IgZ1-F	CACCCAGCATTCTACAGCAAAC	AY643752.1
IgZ1-R	GTCGGTACAAGAACCAAACTCAG	
IgZ2-F	CAGAATGGAGCAAGCCTGAC	EU732710.1
IgZ2-R	TAACTGTGCCCTCTTGGTGTATT	
β-actin-F	ATGGATGAGGAAATCGCTGCC	NM_131031.2
β-actin-R	CTCCCTGATGTCTGGGTCGTC	
